# Malignant Fibrous Histiocytoma of the Breast in Young Male Patient: A Case Report and a Review of the Literature

**DOI:** 10.1155/2013/524305

**Published:** 2013-03-14

**Authors:** Esengul Kocak Uzel, Metin Figen, Tuba Tulin Bek, Kubilay Inanc, Senem Onder, Hazim orhan Kizilkaya

**Affiliations:** ^1^Department of Radiation Oncology, Şişli Etfal Teaching and Research Hospital, 34303 Istanbul, Turkey; ^2^Department of Pathology, Istanbul University Medical Faculty, Turkey

## Abstract

Malignant Fibrous Histiocytoma (MFH) is a fairly common tumor in the deep soft tissues: the most frequent primary sites are the lower (49%) and upper (19%) limbs, but it has been reported even in the retroperitoneum and abdomen (16%), while localization in the breast is extremely rare (1-2). Breast cancer is rarely seen in males, accounts for approximately 1% of all breast cancer, and the breast sarcomas constitute less than 1% of breast tumors in both sexes. In the review of the literature, this is the third male and first young male with MFH. Here, we present a 37-years-old male patient who is diagnosed to have malignant fibrous histiocytoma in a variant of pleomorphic fusiform cell localized in the left breast. Following the wide local excision, the patient was given an adjuvant 50 Gy of external radiotherapy. He remained alive and well after 42 months of followup. We believe that reporting such few cases would contribute to forming treatment algorithms of rare tumors.

## 1. Introduction

Although invasive ductal carcinoma (93.7%) is the most common type of male breast cancer, there are many different types of histologies including papillary (2.6%), mucinous (1.8%), lobular (1.5%), medullary (0.5%), and the breast sarcoma, the latter constitutes less than 1% of all breast tumors in the review of the literature [4–15]. Fibrosarcoma, leiomyosarcoma, angiosarcoma, leiomyosarcoma, osteosarcoma, rhabdomyosarcoma, dermatofibrosarcoma protuberans, and malignant fibrous histiocytoma are the types of sarcomas, which are encountered in breast [15]. Malignant fibrous histiocytoma is a tumor, which is originated from the connective tissues of glands. MFH can occur either primarily or secondarily after radiation exposure [16, 17]. In this paper, a 37-years-old male patient who has a diagnosis of malignant fibrous histiocytoma is presented.

## 2. Case Report

**Figure 1:**
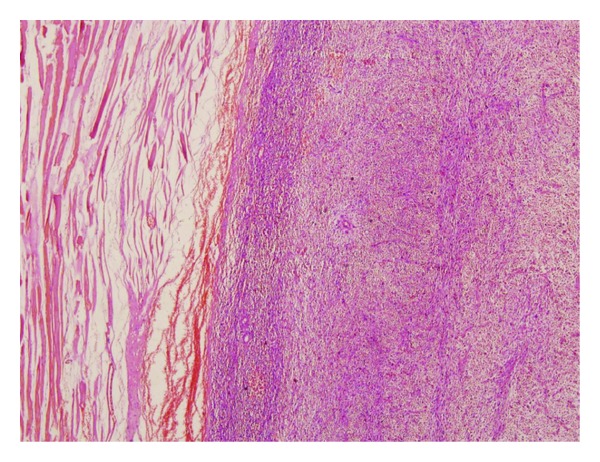
Microscopic image of the biopsy, stained with H&E; storiform pattern indicated malignant fibrous histiocytoma near the pectoral muscle (H&E ×20).

**Figure 2:**
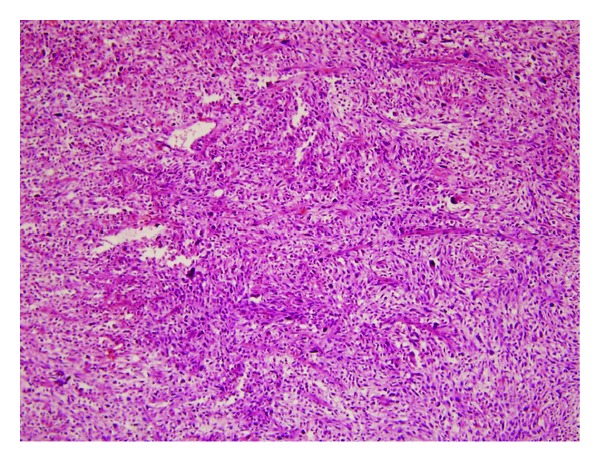
Microscopic image of the biopsy, stained with H&E; epithelioid spindle cells among giant cells in it (H&E ×40).

**Figure 3:**
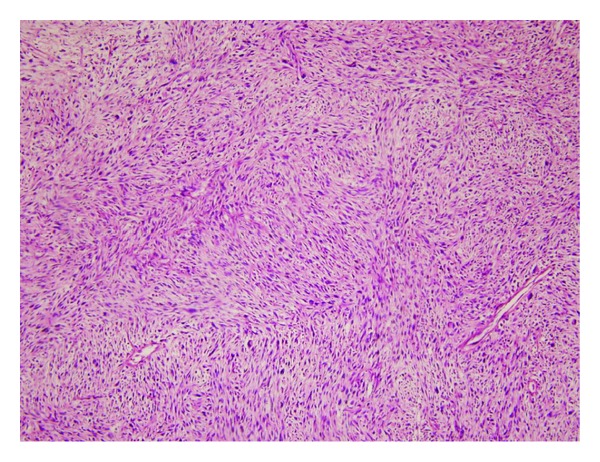
Microscopic image of the biopsy, stained with H&E; spindle cells composing storiform pattern (H&E ×100).

**Figure 4:**
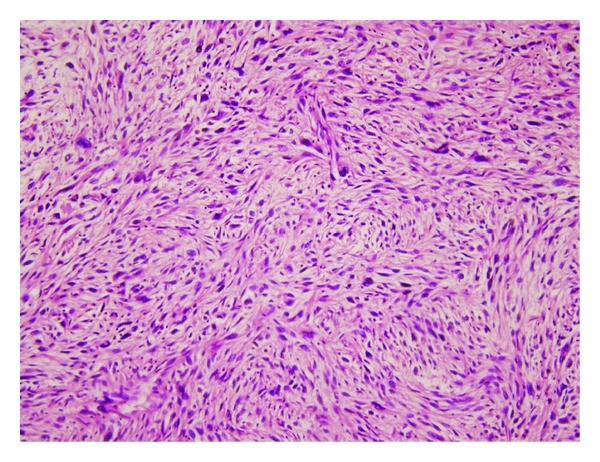
Microscopic image of the biopsy, stained with H&E; spindle cells composing storiform pattern (H&E ×200).

H. A. is a male patient, who has no any known disease and no previous history of radiotherapy for any reason. He presented with a painless mass in his left breast. On examination, there was a mass measuring 12 centimeters in diameter on his left breast. There was no palpable axillary nodes. Following fine-needle aspiration biopsy, a wide local excision of the mass was performed, which is reported as a sarcoma with fusiform cell, and the mass, measuring 12∗9∗8.5 cm, was excised totally. The lesion was found in 0.2 cm distance to anterior and superior surgical margin, and other margins were more than 0.5 cm; no tumor was seen in surgical margins. Immunohistochemical staining and morphological findings were compatible with malignant fibrous histiocytoma, and sarcoma with pleomorphic fusiform cell was reported as subvariant (Figures 1, 2, 3, and 4). There was no distant metastasis on CT scan of the chest, and no adjuvant systemic therapy was suggested to the patient. The patient was offered a postoperative RT because of inadequate surgical margins. A total dose of 50 Gy in 25 fractions with two tangential wedged fields by using 6 MV photons of a linear accelerator was given to his left chest wall. The course radiotherapy was uneventful apart from a mild skin reaction. The follow up of the patient was performed every 3 months for the first year, every 4 months for the second year, and 6 months thereafter. Physical examination was performed at every visit, and CT of thorax was obtained yearly. The patient remained well, and no evidence of local or systemic disease was detected after 42 months of followup.

## 3. Discussion

Malignant fibrous histiocytoma (MFH) is a heterogeneous group of malignant myofibroblastic-fibrohistiocytic tumors, with well-defined storiform-pleomorphic, myxoid, giant cell-rich, and inflammatory morphological variants, [2, 3, 18–21] and our case is placed within pleomorphic fusiform variant. MFH is a tumor rarely seen in breast, and about 50 cases regarding this phenomenon were indicated so far [2, 3, 18–31]. It is identified mostly in middle aged women, and only 3 cases reported in elderly males so far [1–3]. Our determination offers significance in the sense that our patient is the fourth and the youngest male case according to the literature [1, 21–32], and Table 1 summarized the four cases. Adverse prognostic factors in MFH include the size of the tumor, the presence of distant metastasis, and older age [3, 14]. MFH could be rarely aggressive, if so a high rate of local recurrence (44%) and distant metastasis (42%), particularly to the lungs (80%), bony skeleton, pleura, and liver, can occur. Regional lymph nodes involvement ranges from 12% to 32% [1, 32, 33]; however, routine axillary dissection has not been defined [29, 30]. The studies available in the literature report occasional skin and subcutaneous soft tissue metastasis due to MFH usually as a terminal event [1, 31–34]. The main treatment choice for MFH as well as that of sarcoma is surgery. Some researchers indicate that it must be in the form of mastectomy which includes the pectoral muscles in order to reduce the rate of local recurrence [29]. While some others indicate that, with the condition of providing the negative limits, breast preserving surgery would be sufficient for smaller tumors which are less than 5 cm [30]. Despite that the mass in our patient was large, a wide local excision was performed, and with the addition of RT no recurrent lesion was identified so far. No routine axillary dissection is proposed in this case as did some others [14–17, 29]. In the absence of prospective randomized trials, there is still uncertainty as to the role of adjuvant therapy. Adjuvant radiotherapy could be considered, for patients in whom tumor larger than 5 cm, close or positive surgical margins [7, 31]. Adjuvant radiotherapy was added because our patient had inadequate margins. We believe that application of adjuvant radiotherapy has a significant contribution to the local control, therefore, long-term disease-free period. There is still no clearness about chemotherapy while hormonotherapy has no place in MFH [14, 16, 29].

As a conclusion, we believe that the increase in number of cases in the literature will help and contribute to the embodiment of therapeutic algorithm of the disease in question.

## Figures and Tables

**Table 1:** Four patients' characteristic.

The case	Age of the case	Size of tumor	Treatment	Followup—alive (months)
Lunde et al. 1986 [1]	66	NA	Surgery + axillary lymphadenectomy + radiotherapy	18
Mahalingam et al. 2011 [2]	72	3.1 ∗ 2.3 cm	Surgery + radiotherapy	36
Hartel et al. 2011 [3]	67	NA	NA	NA
Our case 2013	37	12 ∗ 9 ∗ 8.5 cm	Surgery + radiotherapy	42

NA: not applicable.
